# Molecular analysis of the *CTSK* gene in a cohort of 33 Brazilian families with pycnodysostosis from a cluster in a Brazilian Northeast region

**DOI:** 10.1186/s40001-016-0228-7

**Published:** 2016-08-24

**Authors:** Thaís Fenz Araujo, Erlane Marques Ribeiro, Anderson Pontes Arruda, Carolina Araujo Moreno, Paula Frassinetti Vasconcelos de Medeiros, Renata Moldenhauer Minillo, Débora Gusmão Melo, Chong Ae Kim, Maria Juliana Rodovalho Doriqui, Têmis Maria Felix, Rodrigo Ambrosio Fock, Denise Pontes Cavalcanti

**Affiliations:** 1 Skeletal Dysplasia Group, Department of Medical Genetics, Faculty of Medical Sciences, State University of Campinas, Campinas, SP Brazil; 2 Children’s Hospital Albert Sabin, Fortaleza, CE Brazil; 3 Medical Sciences Faculty of Juazeiro do Norte (FMJ), Juazeiro do Norte, CE Brazil; 4 Perinatal Genetics Program, Department of Medical Genetics, Faculty of Medical Sciences, State University of Campinas, Campinas, SP Brazil; 5 Federal University of Campina Grande, Campina Grande, PB Brazil; 6 Children’s Clinic, City Hall of Guarulhos, Guarulhos, SP Brazil; 7 Medical Department, Federal University of de São Carlos (UFSCAR), São Carlos, SP Brazil; 8 Medical Genetics Unit, Children’s Institute, Medical Sciences Faculty, University of São Paulo (FCMUSP), São Paulo, SP Brazil; 9 Children’s Hospital Juvêncio Mattos, São Luís, MA Brazil; 10 Medical Genetics Service, Clinical Hospital of Porto Alegre, Porto Alegre, RS Brazil; 11 Centro de Genética Médica da Universidade Federal de São Paulo, São Paulo, SP Brazil

**Keywords:** Pycnodysostosis, Cathepsin K, Inbreeding, Novel mutation

## Abstract

**Background:**

Pycnodysostosis is an autosomal recessive skeletal dysplasia, the prevalence of which is estimated to be low (1 per million). Nevertheless, in recent years we have found 27 affected individuals from 22 families in Ceará State, a region of the Brazilian Northeast, giving a local prevalence of 3 per million. This local prevalence associated with a high parental consanguinity, suggesting a possible founder effect, prompted us to perform a molecular investigation of these families to test this hypothesis.

**Methods:**

The CTSK gene was sequenced by the Sanger method in the patients and their parents. In addition to 18 families from Ceará, this study also included 15 families from other Brazilian regions. We also investigated the origin of each family from the birthplace of the parents and/or grandparents.

**Results:**

We have studied 39 patients, including 33 probands and 6 sibs, from 33 families with pycnodysostosis and identified six mutations, five previously described (c.436G>C, c.580G>A, c.721C>T, c.830C>T and c.953G>A) and one novel frameshift (c.83dupT). This frameshift variant seems to have a single origin in Ceará State, since the haplotype study using the polymorphic markers D1S2344, D1S442, D1S498 and D1S2715 suggested a common origin. Most of the mutations were found in homozygosity in the patients from Ceará (83.3 %) while in other states the mutations were found in homozygosity in half of patients. We have also shown that most of the families currently living outside of Ceará have northeastern ancestors, suggesting a dispersion of these mutations from the Brazilian Northeast.

**Conclusions:**

The high frequency of pycnodysostosis in Ceará State is the consequence of the high inbreeding in that region. Several mutations, probably introduced a long time ago in Ceará, must have spread due to consanguineous marriages and internal population migration. However, the novel mutation seems to have a single origin in Ceará, suggestive of a founder effect.

**Electronic supplementary material:**

The online version of this article (doi:10.1186/s40001-016-0228-7) contains supplementary material, which is available to authorized users.

## Background

Pycnodysostosis (OMIM 265800) (*Online Mendelian Inheritance in Man*) is a rare autosomal recessive disorder, classified in the skeletal dysplasias group with increase of bone density [1]. Besides the altered bone density, the radiological findings include parietal and frontal bossing, delayed closure of sutures and fontanels, obtuse mandibular angle, susceptibility to fractures and acroosteolysis of the distal phalanges [2, 3]. This skeletal dysplasia is characterized by disproportionally short stature associated with a typical clinical phenotype that includes a peculiar facial appearance and hand brachydactyly with short distal phalanges [2, 3]. Hypoplasia of the maxilla, dental crowding, groove palate, nail and clavicle hypoplasia and recurrent respiratory infections are other signs seen in many patients [4, 5]. Lifespan, intelligence and sexual development are often normal [3].

Pycnodysostosis was first described in 1962 by Maroteaux and Lamy [2] but its molecular basis was only elucidated in 1996 by Gelb and collaborators [6]. Studying a large consanguineous Israeli Arab family, these authors found a common genomic region homozygous-by-descent in all 16 individuals affected by pycnodysostosis in the chromosome 1 (1q21) [6]. Among the genes located in this region, the cathepsin K (*CTSK*) was considered a strong candidate because the encoded protein—lysosomal cysteine protease—is highly expressed in osteoclasts [6]. Sequencing of the *CTSK* gene in several individuals with a suggestive phenotype of pycnodysostosis has provided evidence for mutations in this gene [6–26].

**Fig. 1:**
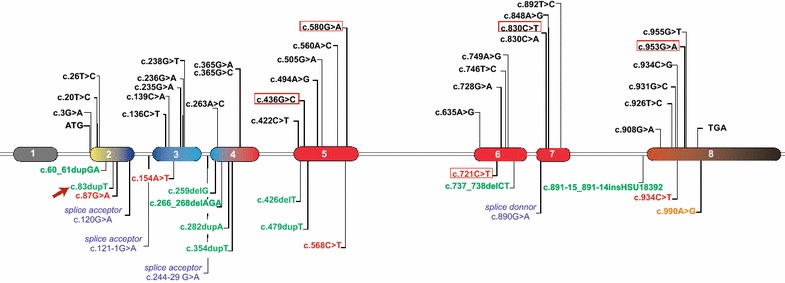
Structure of cathepsin K gene showing the distribution of the 51 reported mutations. There are 31 Missenses mutations (*black*), 9 frameshifts and 1 codon deletion mutations (*green*), 5 nonsense mutations (*red*), 4 splice mutations (*blue*) and 1 stop codon mutation (*orange*). Exon 1 and the final portion of exon 8 are non-coding (*black*). The exons are colored according to region of protein they encode. *Yellow* represents the Pre region (15 amino acids), *blue* indicates the Pro region (99 amino acids) and *red* represents the mature protein region (215 amino acids). The mutations found in the present study are into the *red boxes* and the novel mutation is highlighted with a *red arrow*. For more details about the described mutations see Additional file 2

The *CTSK* gene is approximately 12 kb in size (Transcript ID: ENST00000271651.7) and contains eight exons (GenBank NM_000396.3). The codon for the translation initiator methionine (ATG) is located in exon 2, whereas the termination codon (TGA) is located in the middle of exon 8 [2] (Fig. 1). Due to the potent collagenolytic action in the collagen type I molecule, the major constituent of the bone matrix, the lysosomal cysteine protease, is prominent for its role in the bone remodeling [2, 3].

There is no specific epidemiologic study to estimate the prevalence of pycnodysostosis, but some authors have estimated it to be between 1 and 1.7 per million based on the number of cases described in the literature worldwide [2, 27–29].

In spite of the rarity of pycnodysostosis, in recent years we have found 27 affected individuals from 22 families (59 % consanguineous), all living in Ceará State, a region with a population of 8,904,459 inhabitants located in the Brazilian Northeast, giving a local prevalence of 3 per million. This high frequency of pycnodysostosis prompted us to perform a molecular investigation of the *CTSK* gene in these families to evaluate the hypothesis of a possible founder effect. For this survey, we were able to study a total of 18 families from Ceará and also 15 families from other Brazilian regions. Surprisingly, we found five previously described mutations (c.436G>C, c.580G>A, c.721C>T, c.830C>T, and c.953G>A) and one novel frameshift mutation (c.83dupT), suggesting that the high inbreeding of the families might be the cause of the high prevalence in that region. The observation of the same mutations in other Brazilian regions, also associated with high inbreeding and with northeastern ances*t*ors, suggests a dispersion of these mutations from the Brazilian Northeast. On the other hand, a founder effect might explain the novel mutation.

## Methods

### Patients

A total of 33 families were studied and included 39 patients (33 probands and 6 sibs) and 46 parents. In addition to the clinical and radiological data for each patient, information regarding the birthplace of the respective parents and grandparents was collected. This study had the approval of the Ethics Committee of the State University of Campinas (Comitê de Ética em Pesquisa da Faculdade de Ciências Médicas, Universidade Estadual de Campinas), under the number CEP-671.589, and written informed consent was obtained from each patient and/or from their respective relatives.

### Sequencing analysis

The genomic DNA was extracted by the standard phenol–chloroform protocol from peripheral blood samples obtained from patients and their relatives. The exons and exon–intron boundaries were amplified by polymerase chain reaction (PCR) using primers designed by us (see Additional file 1). PCRs were performed in the presence of 50 ng of genomic DNA, 1× KCl buffer, 0.15 U Taq Polymerase, 200 μM each deoxynucleotide triphosphate (dNTP, Thermo Scientific), 0.5 pmol each primer and water to final volume of 10 μL. The PCR products were visualized on 1.5 % agarose gels. The amplicons produced by PCR were purified and were submitted to the bidirectional direct sequencing using the BigDye Terminator Cycle Sequencing Ready Reaction Kit version 3.1 (Applied Biosystems, Foster City, CA, USA), and were analyzed with an ABI 3500 XL automatic sequencer (Applied Biosystems^®^) using standard methods.

Sequencing data were analyzed through the CodonCodeAligner version 4.1.1 software and the mutations found were compared with online data banks (*Ensembl*, HGMD^®^, NCBI, dbSNP and LOVD^®^). The novel mutation was valued by online prediction mutation software (http://www.mutationtaster.org/). In addition, 100 alleles from 50 control individuals were investigated for the novel mutation.

### Haplotype study

Four FAM-labeled microsatellite markers (Fluorescein Amidite) (D1S2344, D1S442, D1S498 and D1S2715) previously used in other similar studies [11, 29–31] were selected from the literature. The primer sequences and physical distance of the markers to *CTSK* gene were obtained from *Ensembl* (http://www.ensembl.org/index.html). PCR conditions were performed as described in the sequencing analysis and the primers are indicated in Additional file 1. The amplicons were genotyped using Formamida Hi-Di™ (Applied Biosystems^®^), 600 LIZ^®^v2.0 size Standard (Applied Biosystems^®^) and ABI 3500 XL (Applied Biosystems^®^). Genotyping data were analyzed using GeneMapper4.1^®^ software.

## Results

The sequencing analyses from 33 probands, six sibs, and their parents are summarized in Table 1. The parental origin of the allele could be identified, except in the compound heterozygous individuals from cases 25 and 31, since DNA from both parents was unavailable. Recurrence occurred in nine families, but three were unavailable for study.

**Table 1:** Data for the patients reported in the present manuscript: gender, coefficient of inbreeding (*F*), family recurrence, genotype and localization in exons

Case number	Gender	*F*	Rec.	Index case genotype			
				Maternal allele	Exon	Paternal allele	Exon
Ceará State							
1	M	0	N	c.83dupT	2	c.83dupT	2
2	F	0	N	c.83dupT	2	c.721C>T	6
3	M	0	N	c.721C>T	6	c.721C>T	6
4	F	1/32	N	c.953G>A	8	c.953G>A	8
5	F	0	N	c.83dupT	2	c.83dupT	2
6	M	0	N	c.721C>T	6	c.721C>T	6
7	F	?	Sister^a^	c.721C>T	6	c.721C>T	6
8	M	0	Sister	c.436G>C	5	c.436G>C	5
9	M	0	N	c.83dupT	2	c.83dupT	2
10	M	1/256	N	c.83dupT	2	c.83dupT	2
11	M	1/16	N	c.721C>T	6	c.721C>T	6
12	F	0	Brother^b^	c.721C>T	6	c.721C>T	6
13	M	1/16	N	c.436G>C	5	c.436G>C	5
14	M	0	Brother	c.436G>C	5	c.580G>A	5
15	M	1/16	N	c.436G>C	5	c.436G>C	5
16	M	0	N	c.83dupT	2	c.83dupT	2
17	F	0	N	c.83dupT	2	c.721C>T	6
18	M	0	N	c.436G>C	5	c.436G>C	5
Other states							
19	M	?	Sister^a^	c.436G>C	5	c.436G>C	5
20	M	0	Sister	c.721C>T	6	c.436G>C	5
21	F	0	Brother^a^	c.953G>A	8	c.953G>A	8
22	F	0	N	c.953G>A	8	c.721C>T	6
23	M	?	N	c.721C>T	6	c.721C>T	6
24	M	1/16	N	c.721C>T	6	c.721C>T	6
26	F	0	Sister	c.83dupT	2	c.721C>T	6
27	F	1/64	N	c.436G>C	5	c.436G>C	5
28	M	1/256	N	c.953G>A	8	c.953G>A	8
29	M	0	Sister	c.721C>T	6	c.436G>C	5
30	F	0	N	c.953G>A	8	c.83dupT	2
32	M	1/16	N	c.721C>T	6	c.721C>T	6
33	F	1/256	N	c.830C>T	7	c.830C>T	7
				**Allele 1**		**Allele 2**	
25^c^	M	0	N	c.436G>C	5	c.721C>T	6
31^c^	M	0	N	c.436G>C	5	c.721C>T	6

Patients 1–18 are from Ceará State (Brazilian Northeast), Patients 19 to 21 are from Paraíba State (Brazilian Northeast), Patients 22–25 are from Maranhão State (Brazilian Northeast), Patient 26 is from Goiás State (Brazilian Central-west), Patients 27–32 are from São Paulo State (Brazilian Southeast) and Patient 33 is from Rio Grande do Sul State (Brazilian South)

M, male; F, female; Y, yes; N, no; ?, parents are consanguineous but unaware of the family relationship; *F*, coefficient of inbreeding; Rec., recurrence

^a^DNA not available for analysis

^b^No mutations were found

^c^The origin of each allele could not be proven

**Fig. 2:**
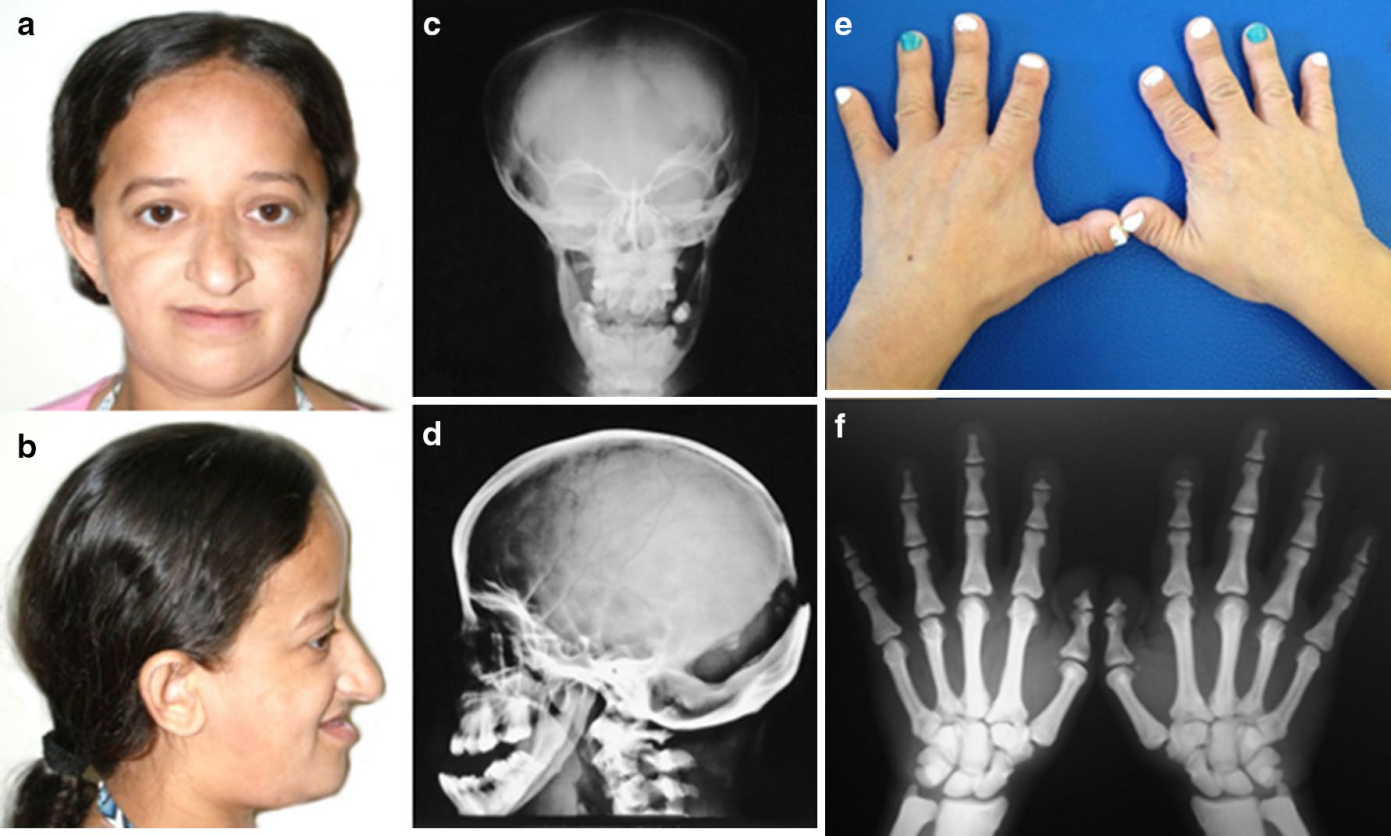
Clinical and radiological findings of two patients affected by pycnodysostosis. **a**–**b** The typical facial appearance of the pycnodysostosis (large frontal, midfacial hypoplasia, low set ears, and beaked nose), here observed in the patient 27. **c**–**d** Cranial X-rays of patient 27 showing increased bone density, opened sutures, parietal bossing and obtuse mandibular angle. **e** The hands of patient 27 showing brachydactyly. **f** X-rays of the hands of the patient 2 showing increased bone density, brachydactyly and acroosteolysis of the distal phalanges, mainly observed in both second fingers

Although the pycnodysostosis phenotype is well known, the main clinical and radiological findings of two patients here studied are shown in Fig. 2.

Parental consanguinity was mentioned by 13 families (39. 4 %). In three of them (cases 7, 19 and 23), the parents recognized they are related but were unaware of their degree of kinship, and were therefore interpreted as having a distant consanguinity (*F* < 1/64) (*F* = coefficient of inbreeding) (Table 1).

### Mutations found in patients from Ceará

**Fig. 3:**
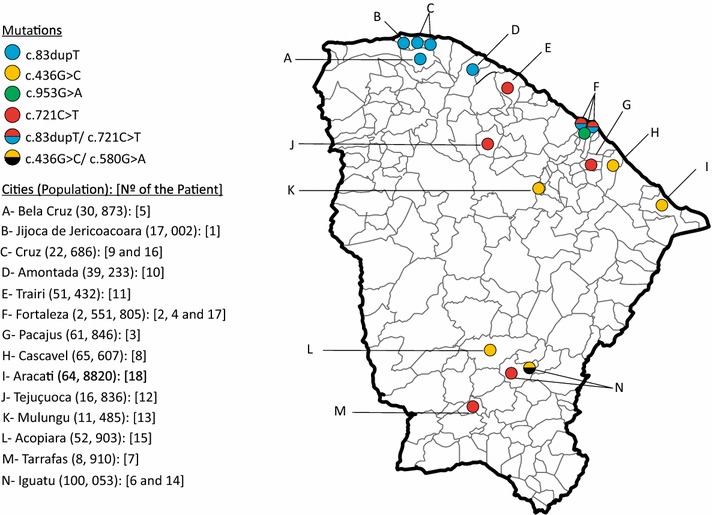
Distribution of mutations found in patients from Ceará (1–16). Each *circle* represents a patient carrying one specific mutation (homozygous) or two (compound heterozygous), according to the *colors* in the caption

Rather than a single mutation, five different mutations were found in Ceará State, four of which have been previously described: c.436G>C (p.G146R), c.580G>A (p.G194S), c.721C>T (p.R241*), c.953G>A (p.C318Y) and a novel one c.83dupT (p.W29Mfs*10) (Table 1). The geographic distribution of mutations in Ceará State shows that the majority are concentrated in small cities (Fig. 3), especially the novel mutation, that seems to have a single origin in a small region on Ceará’s Northwestern coast.

**Fig. 4:**
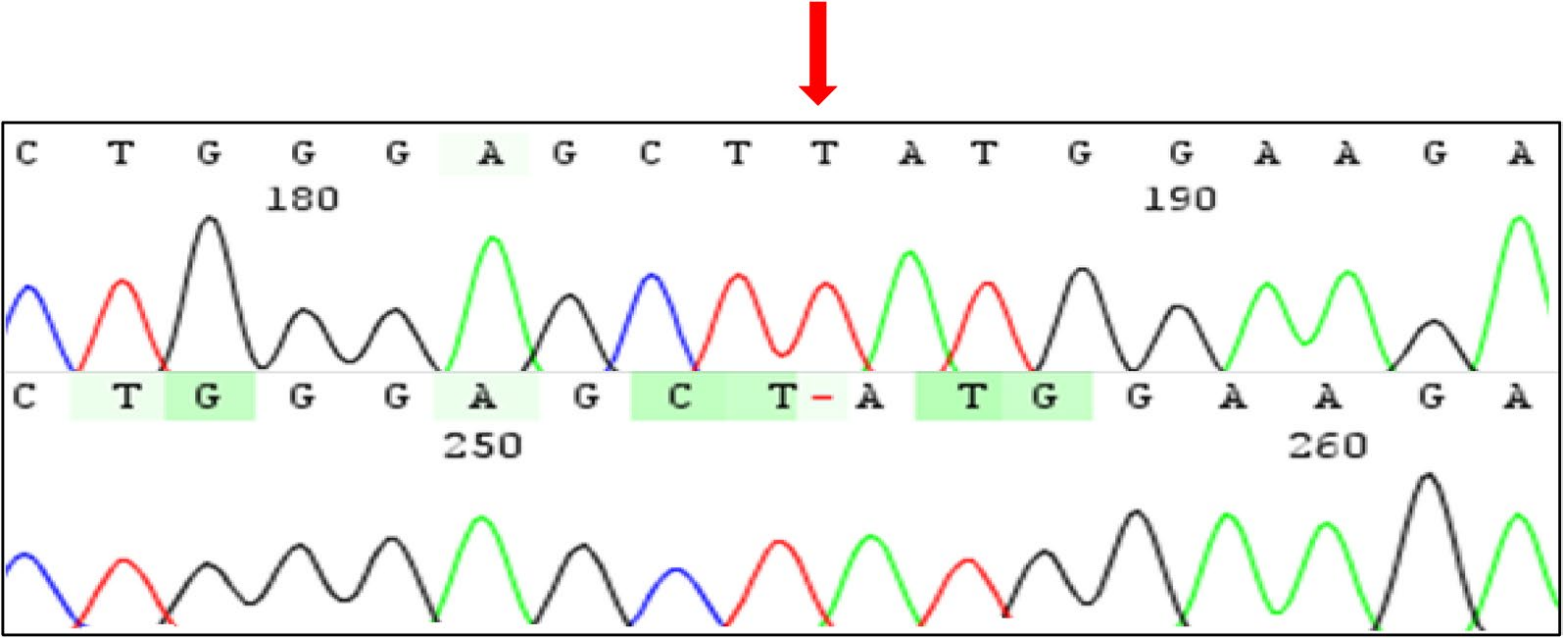
Electropherogram showing a novel frameshift mutation c.83dupT (p.W29Mfs*10) found in Brazilians patients. The *red arrow* indicates a duplication of a thymine in homozygosity found in a patient with pycnodysostosis and below is the sequencing of a control individual

The novel mutation (c.83dupT) is a duplication of a thymine in position 83 in the exon 2 of the cDNA (Fig. 4). This frameshift mutation should impact the protein function, since it disrupts ten amino acids at the end of the exon 2 (W29Mfs*10). As expected, analysis in silico predicted this variation as a pathogenic mutation, and moreover, it was absent in 100 alleles from control individuals. A set of all described mutations is shown in Fig. 1 and Additional file 2.

### Mutations found in patients from other States

**Fig. 5:**
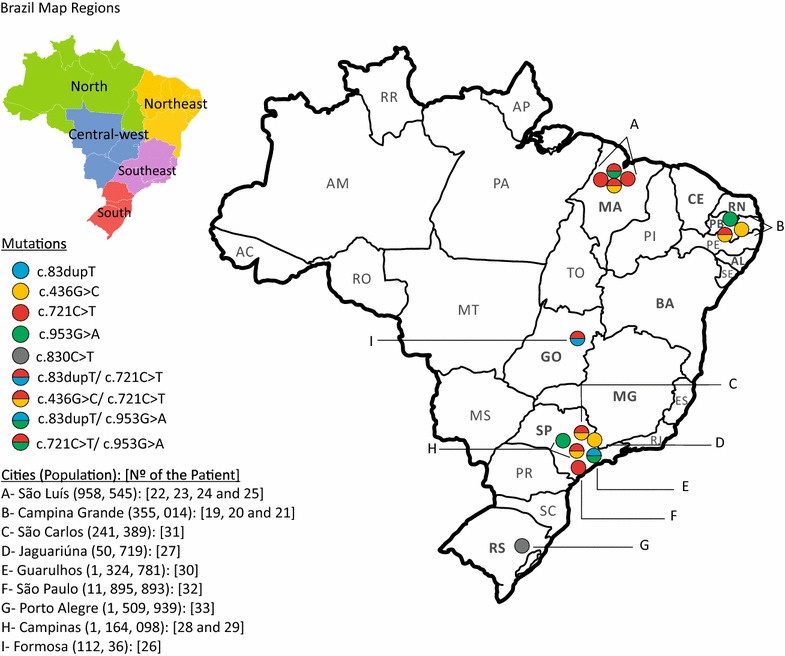
Distribution of mutations according to actual address of patients 19–33. Each *circle* represents a patient carrying one specific mutation (homozygous) or two (compound heterozygous), according to the *colors* in the caption. *AC* Acre, *AL* Alagoas, *AM* Amazonas, *AP* Amapá, *BA* Bahia, *CE* Ceará, *ES* Espírito Santo, *GO* Goiás, *MA* Maranhão, *MG* Minas Gerais, *MS* Mato Grosso do Sul, *MT* Mato Grosso, *PA* Pará, *PB* Paraíba, *PE* Pernambuco, *PI* Piauí, *PR* Paraná, *RJ* Rio de Janeiro, *RN* Rio Grande do Norte, *RO* Rondônia, *RR* Roraima, *RS* Rio Grande do Sul, *SC* Santa Catarina, *SE* Sergipe, *SP* São Paulo, *TO* Tocantins. More information about patients’ origin and precedence is available in Additional file 3

All the mutations found in Ceará, except one (c.580G>A), were also observed in other Brazilian regions (Fig. 5). The c.830C>T mutation was observed in a single family from the South region (Table 1), making a total of six different mutations detected in the complete survey.

When we analyzed the family background regarding the parents and grandparents birthplaces of patients currently living outside Ceará, we found that all but two (31 and 33) have an ancestor coming from the Northeast region, close to Ceará State (see Additional file 3). Patient 31 is from the backlands of São Paulo State and just recognizes local and European ancestor in his family. Patient 33 is from the hinterland of Rio Grande do Sul State and has Italian ancestors.

### Haplotype study

**Fig. 6:**
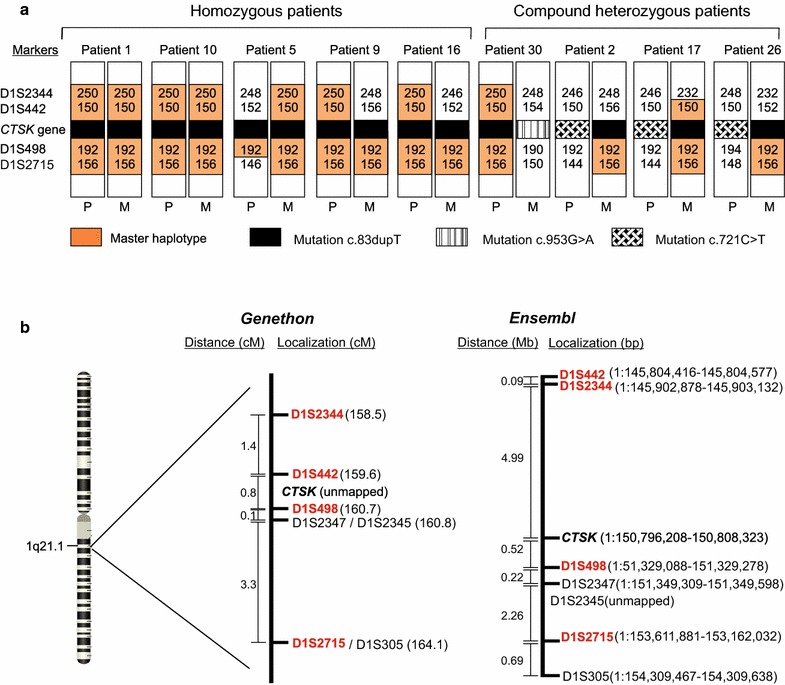
**a** Haplotypes of nine patients with pycnodysostosis carrying the novel frameshift mutation. **b** Localization and distance of markers and *CTSK* gene. The markers used in the present study are highlighted. The cathepsin K (*CTSK*) gene is located between markers D1S442 and D1S498, according to *Ensembl*. *P* paternal allele, *M* maternal allele, *cM* centiMorgan, *Mb* mega base pairs, *bp* base pair

The most frequent mutation in the whole casistic was the c.721C>T (16/33), followed by the c.436G>C (11/33) and the novel mutation c.83dupT (9/33), the latter being concentrated in Ceará state. For this novel mutation, we performed a haplotype study, aiming to test a founder effect hypothesis. Nine patients carrying this novel mutation and their parents were analyzed to identify the haplotype phase. Eight out of 14 chromosomes carrying the c.83dupT mutation also carried a master haplotype of 5.6 cM (according to *Genethon*), whereas all chromosomes but one from Patient 5, carried a preserved piece of the master haplotype, characterized by markers D1S498 and D1S2715 (Fig. 6a). This preserved region in almost all chromosomes from patients with the same mutations strongly suggests that these patients share a common ancestor. Figure 6b shows the genetic and physical localization from markers and *CTSK* gene given by *Genethon* and *Ensembl*, respectively. In this same figure, we can notice that apart from being different the proportional distance between the markers in the two maps, the order of markers D1S2344 and D1S442 has changed.

## Discussion

### Inbreeding population in Ceará State leading to high frequency of pycnodysostosis

Due to the observed high frequency of pycnodysostosis in Ceará State, this survey first focused on the molecular study of patients coming from that region. We have found five different mutations in these patients (c.83dupT, c.436G>C, c.580G>A, c.721C>T, c.953G>A) (Table 1). Although 33.3 % of the families had referred consanguinity, most of the mutations (83.3 %) were found in homozygosity (Table 1), which strongly suggests that this is the real consanguinity rate among these families, probably reflecting a distant consanguinity in some families. Therefore, the initial hypothesis of a founder effect was ruled out and we now propose that the high consanguinity rate is the main cause of the high frequency of pycnodysostosis in this region. The consanguinity rate in the whole Brazilian Northeast is widely recognized as being higher than in other Brazilian regions [32, 33]. While the overall mean value of frequency of consanguineous marriages in Brazil is estimated to be 4.8 %, in Northeastern Brazil this rate varies from 6 to 12 % [34]. According to Freire-Maia, 1957 [33], the mean consanguinity rate in Ceará State is close to 9 %. However, some small and particular areas in the hinterland of the Brazilian Northeast reveal a consanguinity rate as high as 60 % [34].

Consanguineous marriages with the common ancestor(s) placed more distant than *F* ≤ 1/64, at least in theory, would result in an insignificant probability of homozygosity by descent. However, deleterious autosomal recessive alleles may remain hidden in a heterozygous state within some families for many generations, and then consanguineous marriages between mutation carriers, even with distant consanguinity, could cause them to come to the surface [34]. Since the probability of a carrier finding a partner in the general population who bears the same mutation is very small, this effect is more astonishing for rare diseases [35]. Thus, if we examine a group of families affected by proven autosomal recessive diseases, we must find a higher consanguinity rate than in the original population from which these families come [34].

### Novel mutation (c.83dupT, p.W29Mfs*10) and haplotype study

Since the novel mutation was found to be concentrated on the Northwest coast of Ceará State (Fig. 3), we hypothesize this mutation could have been originated or introduced by immigrants in some of those cities and then spread due to consanguineous marriages as a founder effect. The haplotype study showed a master haplotype present in 8 of 14 chromosomes carrying the novel mutation and a preserved region including markers D1S498 and D1S2715 in 13 of 14 chromosomes (Fig. 6a). This reinforces the founder effect hypothesis for this mutation and suggests that the patients carrying the novel mutation have a related common ancestor.

The variations of length found in markers D1S2344 and D1S442 among our patients can be explained by the physical distance of these markers to *CTSK* gene given by *Ensembl*. Whereas marker D1S498 is about 0.5 Mb and the marker D1S2715 is 3 Mb far away from cathepsin K gene, the markers D1S2344 and D1S442 are about 5 Mb distant from this gene (Fig. 6b). The more distant the two *loci* are, the higher the probability of recombination is between them. Although many haplotype studies still use these same markers and consider the genetic localization given by *Genethon* (Fig. 6b), recombination in the first two was also detected by other authors [11, 29, 31].

### Geographic origin and distribution of mutations found in this survey

Unlike the novel mutation, the most frequent mutations c.721C>T and c.436G>C were found more disperse in Ceará State (Fig. 3) and also in other Brazilian regions (Fig. 5), probably suggesting that their dispersion began earlier. The c.721C>T (p.R241*) mutation has already been reported in patients from Portugal [8, 13], Spain [8, 9, 13], Italy [13], Canada [9], and Mexico [31]. The c.436G>C (p.G146R) mutation, however, was identified in patients from Tunisia [25] and Morocco [6]. Moreover, both mutations were already described in other Brazilian patients previously reported, but unfortunately the origin of the patients was not stated [16].

The mutation c.953G>A (C318Y), observed in just one family from Ceará State, was first reported in three Brazilian patients—two in homozygosity and in one case as a compound heterozygous [16]. Recently, it was detected in an African patient [36]. Another mutation, the c.580G>A (p.G194S), described once in a patient living in a region in the South of the Ceará State, was detected in an Italian patient so far [13]. We believe that, unlike the novel mutation c.83dupT, never identified in other patients outside of Brazil, the other mutations (c.436G>C, c.721C>T and c.953G>A) must have been introduced in Northeast Brazil a long time ago, and then spread to other regions due to consanguineous marriages, since most of these patients have ancestors in the Northeast region (see Additional file 3). This is particularly interesting when we look at the novel mutation c.83dupT, which was found in a compound heterozygous state in two patients not living Ceará State (patients 26 and 30). In patient 26, the novel mutation was present in the maternal allele, whose ancestor is from Currais Novos, a city located in Rio Grande do Norte, a neighboring State of Ceará (Fig. 5). In patient 30, the novel mutation was in the paternal allele, whose ancestor is from Ceará State (Additional file 3).

The missense mutation, c.830C>T (p.A277V), observed in homozygosity in a single patient from the southern Brazilian region (Table 1; Fig. 5) was originally described in a patient with Belgian and Algerian background [7] and was then found in Japanese [9, 10, 19] and Pakistani patients [13, 29]. In the family studied here, the ancestors are from Italy. The distant consanguinity (*F* = 1/256) in this family could support the idea that this mutation arises de novo in an ancestor and remained hidden for generations until a consanguineous marriage revealed it, as expected with rare diseases [35]. Regarding this mutation, it is also worth noting that it is described as a *hot spot* because of its localization in the CpG dinucleotides regions [2] and near the catalytic domain of the protein [37].

These results suggest that the majority of the mutations found in the families outside Ceará State were spread to other regions by the internal migration of the population. The internal migration in Brazil is usually motivated by economic reasons, when young people leave rural areas or small towns in search of job opportunities in urban centers or larger cities [38]. Bearing in mind that the Northeast region has the lowest socioeconomic level among the five Brazilian regions, the internal migration usually follows the route from Northeast (with a negative migration number) to Southeast (with positive migration), with São Paulo State as the destination of most of migrants [39].

This internal migration from Northeast to Southeast also increases the consanguinity rate in the latter region [32, 40]. In the present study, we have observed that the real consanguinity rate in families outside Ceará State was also high (46.6 %).

## Conclusions

The results of the present study suggest that the high consanguinity rate (83.3 %) in Ceará State, based on the homozygosity rate found, could be the main cause of high frequency of pycnodysostosis in that State. We also found a novel mutation (c.83dupT), that seems to have a single origin on the Northwestern coast of Ceará State, may have been spreading as a founder effect, since most of the patients carrying this mutation have the same haplotype, including markers D1S498 and D1S2715. Finally, by investigating the origin of the parental families, we suggest that the mutations found in the patients affected by pycnodysostosis outside Ceará State are derived from those found in Ceará State due to the process of internal migration. In these patients, the consanguinity is also high (46.6 %).

## Additional files

10.1186/s40001-016-0228-7 Nucleotide sequence of each primer and PCR product length.
10.1186/s40001-016-0228-7 Summary of all mutations described in cathepsin K gene.
10.1186/s40001-016-0228-7 The geographic origin of mutations according to family background.

## Abbreviations

A: alanine
AC: State of Acre
AL: State of Alagoas
AM: State of Amazonas
AP: State of Amapá
BA: State of Bahia
bp: base pair
C or Cys: cysteine
c.: cDNA localization
cDNA: complementary DNA, synthesized using a molecule of RNAm as template
CE: State of Ceará
CEP: Comitê de Ética em Pesquisa (Ethics Committee)
cM: centiMorgan
CTSK: cathepsin K
D: aspartic acid
D1S442: polymorphic microsatellite marker
D1S498: polymorphic microsatellite marker
D1S2344: polymorphic microsatellite marker
D1S2715: polymorphic microsatellite marker
dbSNP: *single nucleotide polymorphism database*
del: deletion
DNA: deoxyribonucleic acid
dNTP: deoxynucleotide triphosphate
dup: duplication
E: glutamic acid
*Ensembl*: genome browser for vertebrate that supports research in comparative genomics, evolution, sequence variation and transcriptional regulation
ES: State of Espírito Santo
ext: extension
F: phenilalanine
FAM: fluorescein amidite—a synthetic equivalent of fluorescein dye
FCM: Medical Sciences Faculty of Campinas
FMJ: Medical Sciences Faculty of Juazeiro do Norte
FMUSP: Medical Sciences Faculty, University of São Paulo
fs*: frameshift mutation
G: glycine
GO: State of Goiás
H: histidine
HGMD: *Human Gene Mutation Database*
HGVS: *Human Genome Variation Society*
I: isoleucine
IC: inbreeding coefficient, also known as “F” in population genetics
ICF: Informed consent form
ins: insertion
K or Lys: lysine
Kb: kilobase pairs (10^3^ base pair)
KCl: potassium chloride
L: leucine
LOVD: *Leiden Open (source) Variation Database*
M: metionine
MA: State of Maranhão
Mb: mega base pairs (10^6^ base pair)
μL: microliter (10^−6^ liter)
μM: micromoles (10^−6^ mol)
MG: State of Minas Gerais
MgCl_2_: magnesium chloride
MS: State of Mato Grosso do Sul
MT: State of Mato Grosso
N: asparagine
NCBI: *National Center for Biotechnology Information*
ng: nanogram (10^−9^ g)
NM: reference number of human messenger RNA sequencing
NP: reference number of humam protein sequencing
OCD: osteochondrodysplasia
OMIM: *Online Mendelian Inheritance in Man*
p.: localization in protein (amino acid number)
P: proline
PA: State of Pará
PB: State of Paraíba
PCR: polymerase chain reaction
PE: State of Pernambuco
PI: State of Piauí
pMol: picomoles (10^−12^ mol)
PR: State of Paraná
Q: glutamine
R or Arg: arginine
r.: RNAm localization
RJ: State of Rio de Janeiro
RN: State of Rio Grande do Norte
RNAm: ribonucleic acid messenger
RO: State of Rondônia
RR: State of Roraima
RS: State of Rio Grande do Sul
S or Ser: serine
SC: State of Santa Catarina
SE: State of Sergipe
SP: State of São Paulo
T: threonine
TO: State of Tocantins
U: enzyme unit (conversion of 1 micromole of substrate per minute)
UFSCAR: Federal University of de São Carlos
UNICAMP: Universidade Estadual de Campinas (State University of Campinas)
V: valine
W: tryptofan
X or *: termination code
Y: tyrosine
