# Supplementary material for: Molecular analysis of the CTSK gene in a cohort of 33 Brazilian families with pycnodysostosis from a cluster in a Brazilian Northeast region
Source: Eur J Med Res. 2016 Aug 24;21(1):33. doi: 10.1186/s40001-016-0228-7 (PMC4997772; doi:10.1186/s40001-016-0228-7)
Supplement: Supplementary file 1 — 10.1186/s40001-016-0228-7 Nucleotide sequence of each primer and PCR product length. [file 40001_2016_228_MOESM1_ESM.docx]

**Additional file 1**. **Nucleotide sequence of each primer and PCR product length.**

| **Primer** | **Nucleotide sequence (5’-3’)** | **Label** | **Amplicon length (base pair- bp)** |
| --- | --- | --- | --- |
| **2F** | GTCCTTGGAACCAGATGTAC | - | 417 |
| **2R** | CATGAGTTAGGGAAGAGGG | - |  |
| **34F** | CTCTTCTTGCAGGCTCTTAATTC | - | 636 |
| **34R** | GATGTACCTTAATTCCTTGCCC | - |  |
| **5F** | CAACAGATATTAAGCGGAAGAG | - | 518 |
| **5R** | CTTCTTGGCCTTCTGTGG | - |  |
| **67F** | CTGCTGCCTCTGTTAGTTCAC | - | 744 |
| **67R** | GGGACAGAGAAAGGAATATCG | - |  |
| **8F** | CTTTCCCTCCTTTGATACCTAC | - | 448 |
| **8R** | GGAAGGATCATTTGAAGCAC | - |  |
| **D1S2344F** | TCATGGGACTCTCCATCA | FAM | 231 – 257 |
| **D1S2344R** | AAATACTCAGGAAATGGCCTA | - |  |
| **D1S442F** | AACAAAGCTGGACTGGTAATC | FAM | 162 |
| **D1S442R** | CAGTGTCACACAACTGGTTG | - |  |
| **D1S498F** | TTGCTGAAGGGACATAGTG | FAM | 183-205 |
| **D1S498R** | TGCTGGGTTATATCCAATATC | - |  |
| **D1S2715F** | CACAGGATTCTGCGTCTAACT | FAM | 150-168 |
| **D1S2715R** | TGCTCCAAGAACTGAAGTGA | - |  |

F: *primer forward*, R: *primer reverse.* The first ten primers were named according to exon flanked and were designed using the transcript ID [ENST00000271651](http://www.ensembl.org/Homo_sapiens/Transcript/Summary?db=core;g=ENSG00000143387;r=1:150796208-150808323;t=ENST00000271651) (*Ensembl*) and the following software: Gene Runner, Primer 3 and Primer-BLAST. The other primers were obtained direct from *Ensembl* and the forward ones were labeled with FAM at 5’upstream.
