# Supplementary material for: Molecular analysis of the CTSK gene in a cohort of 33 Brazilian families with pycnodysostosis from a cluster in a Brazilian Northeast region
Source: Eur J Med Res. 2016 Aug 24;21(1):33. doi: 10.1186/s40001-016-0228-7 (PMC4997772; doi:10.1186/s40001-016-0228-7)
Supplement: Supplementary file 2 — 10.1186/s40001-016-0228-7 Summary of all mutations described in cathepsin K gene. [file 40001_2016_228_MOESM2_ESM.docx]

### Additional file 2. Summary of all mutations described in Cathepsin K gene. The table also shows the origin of each mutation in DNA and the effect on amino acid. It is also shown the origin of the first patient described as carrying the mutation and the corresponding author who reported the case. The nomenclature is given according to HGVS ([*Human Genome Variation Society*](http://www.hgvs.org/)) recommendations. This is different from the original nomenclature, which is shown in the last column.

| **Location**  **in DNA** | **Patient’s origin** | **Reference article** | **Coding DNA variant sequence (c.)**  **[RefSeq: NM_000396.3]** | **Effect on aminoacid (p.)**  **[RefSeq:** [**NP_000387**](http://www.ncbi.nlm.nih.gov/nuccore/NP_000387)**.1)** | **Described in the reference article as** |
| --- | --- | --- | --- | --- | --- |
| ***Missense Mutations (31)*** | |  |  |  |  |
| Exon 2  Exon 2  Exon 2  Exon 3  Exon 3  Exon 3  Exon 3  Exon 3  Exon 4  Exon 4  Exon 4  Exon 5  Exon 5  Exon 5  Exon 5  Exon 5  Exon 5  Exon 6  Exon 6  Exon 6  Exon 6  Exon 7  Exon 7  Exon 7  Exon 7  Exon 8  Exon 8  Exon 8  Exon 8  Exon 8  Exon 8 | Turkey  Italy  Japan  Germany  Caribbean  Austria  Northern Europe  Turkey  Bangladesh  Japan  China  Unknown  Morocco, Hispanic  Italy  Turkey  China  Italy  Spain  Pakistan  Spain  Pakistan  Belgium  India, Portugal  Unknown  Japan  Mexico  Denmark  Swiss  Honduras  Brazil  Pakistan | Arman, *et al*.,2014  Donnarumma, *et al*.,2007  Nishi, *et al*., 1999  Schilling, *et al*., 2007  Pangrazio, *et al*., 2014  Fratzl-Zelman, *et al*.,2004  Hou, *et al*., 1999  Arman, *et al*.,2014  Pangrazio *et al.,* 2014  Matsushita, *et al*., 2011  Zheng, *et al*., 2013  Chavassieux, *et al*.,2008  Gelb *et al*., 1996  Donnarumma*, et al*.,2007  Arman, *et al*.,2014  Li, *et al.,* 2009  Donnarumma, *et al*.,2007  Hou, *et al*., 1999  Khan, *et al*., 2010  Donnarumma, *et al*.,2007  Donnarumma, *et al*.,2007  Gelb, *et al*., 1998  Hou, *et al*., 1999  Xue *et al*., 2015  Nishi, *et al*., 1999  Toral-Lopez *et al*.,2010  Haagerup, *et al*.,2000  Nishi, *et al*., 1999  Hou, *et al*., 1999  Bertola *et al*., 2010  Donnarumma, *et al*.,2007 | 3G>A  20T>C  26T>C  136C>T  139C>A  235G>A  236G> A  238G>T  263A>C  365G>C  365G>A  422C>T 436G>C  494A>G  505G>A  560A>C  580G>A  635A>G  728G>A  746T>C  749A>G  830C>T  830C>A  848A>G  892T>C  908G>A  926T>C  931G>C  934C>G  953G>A  955G>T | M1I  L7P  L9P  R46W  R47S  G79R  G79E  D80Y  Q88P  R122P  R122Q  A141V  G146R  Q165R  D169N  Q187P  G194S  Y212C  G243E  I249T  D250G  A277V  A277E  Y283C  W298R  G303E  L309P  A311P  R312G  C318Y  G319C | -  -  -  -  Arg47Ser  340G>A  -  -  -  489G>C  -  546C>T  -  -  -  684A>C  -  -  -  -  935C>T  935C>A  -  -  -  -  -  1039C>G  -  - |
| ***Nonsense Mutations (5)*** | |  |  |  |  |
| Exon 2  Exon 3  Exon 5  Exon 6  Exon 8 | Unknown  Northern Europe  Northern Europe  Hispanic America  Turkey | Xue et al., 2015  Hou, *et al*., 1999  Hou, *et al*., 1999  Gelb, *et al*., 1996  Arman, *et al*.,2014 | 87G>A  154A>T  568C>T  721C>T  934C>T | W29*  K52*  Q190*  R241*  R312* | W29X  K52X  Q190X  R241X  R312X |
| ***Frameshift Mutations (9)*** | |  |  |  |  |
| Exon 2  Exon 2  Exon 4  Exon 4  Exon 4  Exon 5  Exon 5  Exon 6  *Intron 7* | Morocco  Brazil  Austria  Pakistan  Turkey  Japan  India  Caribbean  Turkey | Donnarumma*, et al*.,2007  Araujo *et al*., 2016  Fratzl-Zelman*, et al*.,2004  Donnarumma, *et al*.,2007  Özdemir, *et al*., 2013  Fujita, *et al*., 2000  Singh et al., 2014  Pangrazio, *et al*., 2014  Arman *et al*.,2014 | 60_61dupGA  #^1^ 83dupT  259delG  282dupA  354dupT  426delT  479dupT  737_738delCT  #^2^ 891-15_891-14 ins HSU18392 | I21Rfs*29  W29Mfs*10  V87Ffs*4  V95Sfs*9  V119Cfs*25  F142Lfs*19  L160Ffs*14  S246Cfs*4  ----------------------- | I21RfsX29  -  del363G  S95VfsX9  V119CfsX25  531delT  L160FfsX173  Ser246CysfsX4  N296fX54 |
| ***Codon deletion (1)*** | |  |  |  |  |
| Exon 4 | Pakistan | Pangrazio, *et al*., 2014 | 266_268delAGA | K89del | Lys89del |
| ***Splicing Mutations (4)*** | |  |  |  |  |
| Exon 2  *Intron2*  *Intron 3*  Exon 7 | Thailand  Denmark  Saudi Arabia  Egypt | Utokpat *et al*., 2013  Haagerup, *et al*.,2000  Alhashem et al., 2015  Donnarumma, *et al*.,2007 | #^3^ 120 G>A  121-1G>A  #^4^ 244 -29 G>A  890G>A | ----------------------  V41_M81del  ----------------------  G262Afs*70 | Skip of exon 2  41V_81Mdel  -  r.785_890del |
| ***Stop Codon Mutation (1)*** | |  |  |  |  |
| Exon 8 | Israel | Gelb, *et al*., 1996 | 990A>G | *330Wext*19 | X330W |

Hispanic (Hispanic America): America colonized by Spaniards, #^1^ Present study, #^2^ It was reported that the insertion in intron 7 is an Alu sequence of 301bp inserted in reverse orientation that introduces a new potential splice acceptor site, #^3^ There is no functional study, but the deletion seems to lead the skip of entire exon 2, #^4^ There is no functional study of this mutation so far, but was described that the mutation seems to affect the splice acceptor site in intron 3.
