# Supplementary material for: Molecular analysis of the CTSK gene in a cohort of 33 Brazilian families with pycnodysostosis from a cluster in a Brazilian Northeast region
Source: Eur J Med Res. 2016 Aug 24;21(1):33. doi: 10.1186/s40001-016-0228-7 (PMC4997772; doi:10.1186/s40001-016-0228-7)
Supplement: Supplementary file 3 — 10.1186/s40001-016-0228-7 The geographic origin of mutations according to family background. [file 40001_2016_228_MOESM3_ESM.docx]

**Additional file 3. The geographic origin of mutations according to family background.**

| **Case** | | **Current patients’** | | | **Maternal** | | **Maternal family origin** | | | | | | | | | **Paternal** | **Paternal family origin** | | | | | | |  |
| --- | --- | --- | --- | --- | --- | --- | --- | --- | --- | --- | --- | --- | --- | --- | --- | --- | --- | --- | --- | --- | --- | --- | --- | --- |
| **number** | | **address** | | | **allele** | | **Mother** | | | **Maternal Grandmother** | | | | **Maternal Grandfather** | | **allele** | **Father** | | **Paternal Grandmother** | | **Paternal Grandfather** | | |  |
|  | |  | | |  | | **City State** | | | **City State** | | | | **City State** | |  | **City State** | | **City State** | | **City State** | | |  |
|  | **Ceará State** | |  |  | |  | |  | | |  |  |  |  |  |  |  |  |  |  | |  | |  |
| 1 | | | J.Jericoacoara  Fortaleza  Pacajus  Fortaleza  Bela Cruz  Iguatu  Tarrafas  Cascavel  Cruz  Amontada  Trairi  Tejuçuoca  Mulungu  Iguatu  Acopiara  Cruz | | | c.83dupT | | Cruz | CE | | | Cruz | | CE | Cruz | CE | c.83dupT | J.Jericoacoara | CE | J.Jericoacoara | CE | J. Jericoacoara | CE | |
| 2 | | |  |  |  | c.83dupT | | Itarema | CE | | | Itarema | | CE | Itarema | CE | c.721C>T | Itarema | CE | Itarema | CE | Itarema | CE | |
| 3 | | |  |  |  | c.721C>T | | Pacajus | CE | | | Fortaleza | | CE | Iguatu | CE | c.721C>T | Jaguaretama | CE | Jaguaretama | CE | Jaguaretama | CE | |
| 4 | | |  |  |  | c.953G>A | | Fortaleza | CE | | | - | | - | - | - | c.953G>A | - | CE | - | - | - | - | |
| 5 | | |  |  |  | c.83dupT | | Bela Cruz | CE | | | Bela Cruz | | CE | Bela Cruz | CE | c.83dupT | Bela Cruz | CE | Bela Cruz | CE | Bela Cruz | CE | |
| 6 | | |  |  |  | c.721C>T | | Iguatu | CE | | | - | | PB | - | PB | c.721C>T | Iguatu | CE | Iguatu | CE | Iguatu | CE | |
| 7 | | |  |  |  | c.721C>T | | - | CE | | | - | | - | - | - | c.721C>T | - | CE | - | - | - | - | |
| 8 | | |  |  |  | c.436G>C | | Cascavel | CE | | | Cascavel | | CE | Cascavel | CE | c.436G>C | Mossoró | RN | Cascavel | CE | Mossoró | RN | |
| 9 | | |  |  |  | c.83dupT | | Cruz | CE | | | Cruz | | CE | Cruz | CE | c.83dupT | Cruz | CE | - | - | - | - | |
| 10 | | |  |  |  | c.83dupT | | - | CE | | | - | | - | - | - | c.83dupT | - | CE | - | - | - | - | |
| 11 | | |  |  |  | c.721C>T | | - | CE | | | - | | - | - | - | c.721C>T | - | CE | - | - | - | - | |
| 12 | | |  |  |  | c.721C>T | | - | CE | | | - | | - | - | - | c.721C>T | - | CE | - | - | - | - | |
| 13 | | |  |  |  | c.436G>C | | - | CE | | | - | | - | - | - | c.436G>C | - | CE | - | - | - | - | |
| 14 | | |  |  |  | c.436G>C | | - | CE | | | - | | - | - | - | c.580G>A | - | CE | - | - | - | - | |
| 15 | | |  |  |  | c.436G>C | | - | CE | | | - | | - | - | - | c.436G>C | - | CE | - | - | - | - | |
| 16 | | |  |  |  | c.83dupT | | Cruz | CE | | | - | | - | - | - | c.83dupT | Bela Cruz | CE | - | - | - | - | |
| 17 | | | Fortaleza | | | c.83dupT | | Natal | RN | | | Natal | | RN | Natal | RN | c.721C>T | Fortaleza | CE | Trairi | CE | Fortaleza | CE | |
| 18 | | | Aracati | | | c.436G>C | | Aracati | CE | | | - | | - | - | - | c.436G>C | Aracati | CE | - | - | - | - | |
|  | | | **Other States** | | | |  | |  | | |  | |  |  |  |  |  |  |  |  |  |  | |
| 19 | | | C. Grande | PB | | c.436G>C | | Jericó | PB | | | - | | - | - | - | c.436G>C | Jericó | PB | - | - | - | - | |
| 20 | | | C. Grande | PB | | c.721C>T | | Mombaça | CE | | | - | | - | - | - | c.436G>C | Sousa | PB | - | - | - | - | |
| 21 | | | C. Grande | PB | | c.953G>A | | Arara | PB | | | - | | - | - | - | c.953G>A | Casserengue | PB | - | - | - | - | |
| 22 | | | São Luís | MA | | c.953G>A | | São Luís | MA | | | Grajaú | | MA | Grajaú | MA | c.721C>T | São Luís | MA | Gov.Eug.Barros | MA | I. Carrapatal | MA | |
| 23 | | | São Luís | MA | | c.721C>T | | - | MA | | | - | | - | - | - | c.721C>T | - | MA | - | - | - | - | |
| 24 | | | São Luís | MA | | c.721C>T | | Jenipapolândia | MA | | | - | | - | - | - | c.721C>T | - | MA | - | - | - | - | |
| 25 | | | São Luís | MA | | c.721C>T (?) | | - | MA | | | - | | - | - | - | c.436G>C (?) | - | MA | - | - | - | - | |
| 26 | | | Formosa | GO | | c.83dupT | | Currais Novos | RN | | | Currais Novos | | RN | - | RN | c.721C>T | Uberlândia | MG | Uberlândia | MG | Uberlândia | MG | |
| 27 | | | Jaguariúna | SP | | c.436G>C | | Acopiara | CE | | | - | | - | - | - | c.436G>C | Acopiara | CE | - | - | - | - | |
| 28 | | | Campinas | SP | | c.953G>A | | Arapiraca | AL | | | - | | AL | - | AL | c.953G>A | Feira Grande | AL | Feira Grande | AL | Feira Grande | AL | |
| 29 | | | Campinas | SP | | c.721C>T | | R. Pombal | BA | | | - | | BA | - | BA | c.436G>C | R. Pombal | BA | - | BA | - | BA | |
| 30 | | | Guarulhos | SP | | c.953G>A | | V. Conquista | BA | | | V. Conquista | | BA | V. Conquista | BA | c.83dupT | S. Parnaíba | SP | J. do Norte | CE | - | PR | |
| #31 | | | São Carlos | SP | | c.721C>T (?) | | São Carlos | SP | | | São Carlos | | SP | São Carlos | SP | c.436G>C (?) | São Carlos | SP | São Carlos | SP | São Carlos | SP | |
| 32 | | | São Paulo | SP | | c.721C>T | | Lagoa | PB | | | - | | - | - | - | c.721C>T | Lagoa | PB | - | - | - | - | |
| #33 | | | P. Alegre | RS | | c.830C>T | | Vacaria | RS | | | Vacaria | | RS | Vacaria | RS | c.830C>T | Vacaria | RS | Vacaria | RS | Vacaria | RS | |

# Patient without Northeastern ancestors, (?)Origin of the parental alleles was not proved.

States- AL: Alagoas, BA: Bahia, CE: Ceará, GO: Goiás, MA: Maranhão, MG: Minas Gerais, PB: Paraíba, RN: Rio Grande do Norte, RS: Rio Grande do Sul, SP: São Paulo. Cities- Gov.Eug.Barros: Governador Eugênio de Barros , I. Carrapatal: Ilha de Carrapatal, J. Jericoacoara: Jijoca de Jericoacoara, J. do Norte: Juazeiro do Norte , P. Alegre: Porto Alegre, R.Pombal: Ribeira do Pombal, S. Parnaíba: Santana de Parnaíba, V. Conquista: Vitória da Conquista.
